# RNA around the clock – regulation at the RNA level in biological timing

**DOI:** 10.3389/fpls.2015.00311

**Published:** 2015-05-05

**Authors:** Christine Nolte, Dorothee Staiger

**Affiliations:** Molecular Cell Physiology, Faculty of Biology, Bielefeld UniversityBielefeld, Germany

**Keywords:** circadian oscillation, RNA-binding protein, post-transcriptional regulation

## Abstract

The circadian timing system in plants synchronizes their physiological functions with the environment. This is achieved by a global control of gene expression programs with a considerable part of the transcriptome undergoing 24-h oscillations in steady-state abundance. These circadian oscillations are driven by a set of core clock proteins that generate their own 24-h rhythm through periodic feedback on their own transcription. Additionally, post-transcriptional events are instrumental for oscillations of core clock genes and genes in clock output. Here we provide an update on molecular events at the RNA level that contribute to the 24-h rhythm of the core clock proteins and shape the circadian transcriptome. We focus on the circadian system of the model plant *Arabidopsis thaliana* but also discuss selected regulatory principles in other organisms.

## Introduction

### Regulation of the Flow of Genetic Information at the RNA Level

**FIGURE 1:**
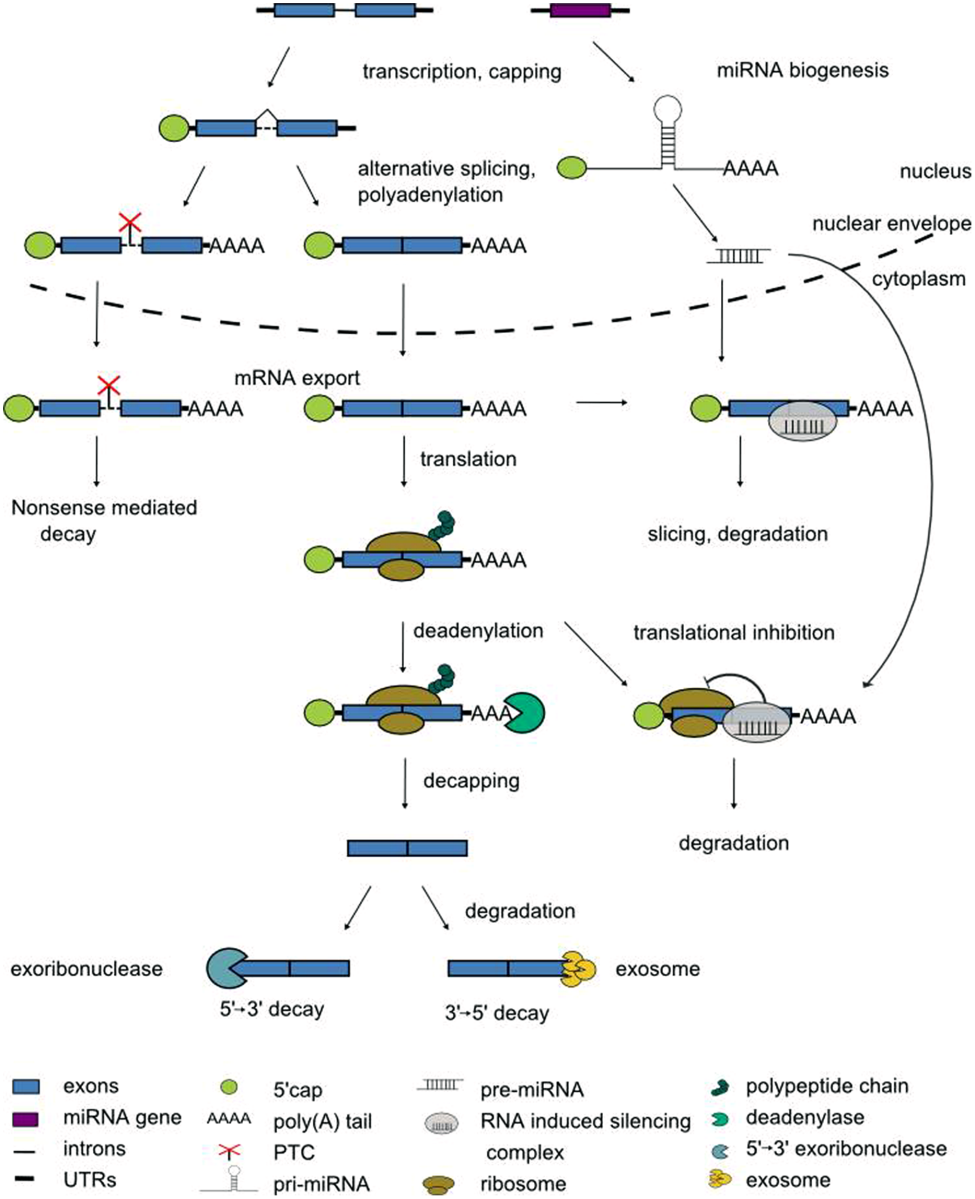
**Steps in pre-mRNA processing. See text for details**.

The expression of eukaryotic genes is regulated at multiple levels. In the nucleus, transcription factors recruit RNA polymerase II to the gene’s promoter. The access of transcriptional activators or repressors to the DNA in turn is licensed by chromatin remodeling factors. Once transcription has been initiated, pre-mRNAs enter a series of processing steps to mature mRNAs (Darnell, 2013). When the nascent pre-mRNA is ca. 20 nucleotides in length, its 5′ end receives the 7-methylguanosine cap to protect the mRNA against degradation. Pre-mRNAs are spliced in the nucleus to remove introns, and differential usage of splice sites can give rise to multiple alternatively spliced transcript isoforms of one and the same pre-mRNA (Syed et al., 2012; Kornblihtt et al., 2013; Reddy et al., 2013). Polyadenylation signals determine the processing at the 3′ end, i.e., the cleavage at a specific position before addition of the poly(A) tail that protects against degradation from the 3′ end (Proudfoot, 2011). Subsequently, the mature mRNA is exported from the nucleus for translation (**Figure 1**).

During this journey from the site of transcription in the nucleus to the cytoplasm the fate of the mRNAs is influenced by two main types of *trans*-acting regulators, RNA-binding proteins and non-coding RNAs. RNA-binding proteins, through dedicated domains, specifically recognize *cis*-active motifs in mRNAs to regulate their processing. The most frequent type of RNA-binding domain is the RNA recognition motif (RRM) of approximately 80 amino acids with a well conserved structure of four antiparallel β strands packed against two α helices (Maris et al., 2005). Non-coding RNAs (ncRNAs) do not have a protein-coding potential. Numerous ncRNAs have emerged as important regulators of gene expression through the identification of cognate mRNA targets via base-pairing to sites with a variable degree of complementarity (Cech and Steitz, 2014). Non-coding RNAs are generally classified according to their size. Long non-coding RNAs (lncRNAs) vary in length between ca. 200 nucleotides and dozens of kilobases (Kim and Sung, 2012). Small non-coding RNAs are between 20 and 25 nucleotides in length and are further classified according to their mode of biogenesis. Small interfering RNAs (siRNAs) are derived from double-stranded precursors whereas microRNAs (miRNAs) are derived from transcripts with partially double-stranded, hairpin-like structures (**Figure 1**; Rogers and Chen, 2013). Collectively, the pre-mRNA processing steps equip the cell with obvious checkpoints to rapidly modulate the transcriptome.

A prominent example of gene expression programs that continuously require fine tuning are periodic fluctuations in mRNA steady-state abundance across the day. Such daily rhythms in gene expression are controlled by an endogenous timing mechanism, the “circadian” clock. The circadian clock acts predominantly by directing promoter activity to defined time intervals of the day. However, off-switching of promoter activity is in many cases not sufficient to account for a steep decline from peak transcript levels to trough levels within a few hours to maintain correct phase, period and amplitude of transcript oscillations.

### The Circadian Timing System

Plant physiology, biochemistry and behavior are orchestrated by the circadian system which serves to optimally align metabolic functions of the plant with the periodic changes in ambient light and dark phases (Barak et al., 2000; Eriksson and Millar, 2003; McClung, 2006; de Montaigu et al., 2010; Yerushalmi et al., 2011). Conceptually, the circadian timing system is divided into three functional units. The core clockwork is responsible for self-sustained 24-h rhythms of clock proteins. Input pathways ensure synchrony of the core clockwork with the day/night cycles through perception of periodic changes in light and temperature. Output pathways drive the expression of a large part of the circadian transcriptome with around a third of the protein-coding genes regulated by the circadian clock (Covington et al., 2008; Hazen et al., 2009). These gene expression rhythms translate into physiological and biochemical output rhythms.

**FIGURE 2:**
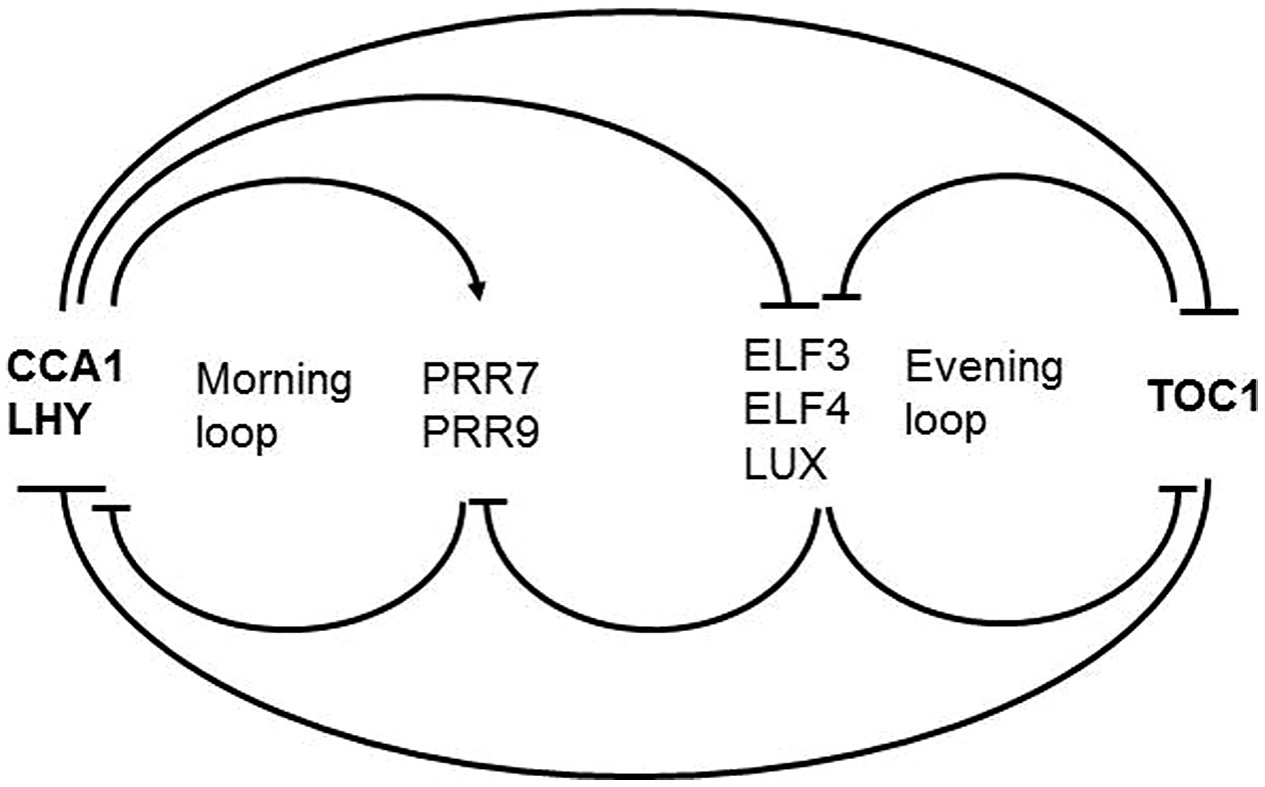
**Scheme of the *Arabidopsis* core clockwork**. In the central loop LHY and CCA1 on the one hand and TOC1 on the other hand reciprocally repress their own expression. In the morning loop, LHY and CCA1 activate *PRR9* and *PRR7* which in turn repress *CCA1* and *LHY*. *PRR7* and *PRR9* expression is switched off during the night through the EC consisting of LUX, EFL3, and ELF4. In the evening loop, the EC and TOC1 reciprocally regulate their expression.

The framework of the plant circadian timekeeping system has been established in *Arabidopsis thaliana.* Below we describe the components that are relevant for the topic we cover here. For a complete picture readers are referred to dedicated reviews (Yanovsky and Kay, 2001; Staiger, 2002; Harmer, 2009; McClung, 2011; Herrero and Davis, 2012; Nagel and Kay, 2012; Staiger et al., 2013; Hsu and Harmer, 2014). The core clockwork is made up by a series of autoregulatory circuits of clock proteins (**Figure 2**). The central loop consists of two Myb transcription factors LATE ELONGATED HYPOCOTYL (LHY) and CIRCADIAN CLOCK ASSOCIATED 1 (CCA1) peaking at dawn, and the pseudoresponse regulator TIMING OF CAB EXPRESSION1 (TOC1) peaking at dusk, that reciprocally repress their own expression (Alabadi et al., 2001; Gendron et al., 2012; Huang et al., 2012). This core loop is interconnected with a loop preferentially active in the morning and another loop preferentially active in the evening. Through the morning loop, LHY and CCA1 activate the expression of the PSEUDORESPONSE REGULATORS *PRR9* and *PRR7* which in turn repress *CCA1* and *LHY*. *PRR7* and *PRR9* expression is switched off during the night through the evening complex (EC) consisting of the Myb-type transcription factor LUX ARRHYTHMO (LUX), EARLY FLOWERING 3 (EFL3) and ELF4 proteins (Dixon et al., 2011; Helfer et al., 2011; Herrero et al., 2012). As a result, *CCA1* and *LHY* transcription resumes. In the evening loop, the EC and TOC1 reciprocally regulate their expression. The interconnection of these feedback loops is thought to contribute to robustness of the rhythmic expression patterns.

Modification of clock proteins, time-of-day dependent proteolysis and regulated nuclear uptake contribute to maintaining a period of 24 h (Mas et al., 2003; Fujiwara et al., 2008; Wang et al., 2010). These post-translational processes operating at the level of clock proteins are beyond the scope of this manuscript and have been reviewed elsewhere (Schöning and Staiger, 2005; Mehra et al., 2009; Seo and Mas, 2014).

Rhythms in eukaryotes generally arise in feedback loops of clock gene transcription/translation. The molecular players, however, are different in plants, arguing for an independent origin of plant and animal clocks (Roden and Carré, 2001). Below, we briefly touch upon the core components of the mammalian and fungal timing systems.

In mammals, the core clock loop comprises two transcription factors, CLOCK and BMAL1 that activate the *Period* (*Per*) *1*, *2,* and *3* and *Cryptochrome* (*Cry*) *1* and *2* genes (Young and Kay, 2001; Dibner et al., 2010; Partch et al., 2013). PER and CRY proteins undergo heterodimerization and are taken up into the nucleus to inhibit activation of their own genes by CLOCK and BMAL1. Upon proteoloytic degradation of CLOCK and BMAL1 a new cycle can then be initiated.

In *Neurospora crassa*, the transcription factors White collar 1 (WC-1) and WC-2 form the white collar complex (WCC), which drives the rhythmic expression of the *frequency* (*frq*) clock gene (Liu et al., 1999; Bell-Pedersen et al., 2005). FRQ interacts with frequency interacting RNA helicase (FRH). The resulting FRQ/FRH complex in turn inhibits WCC activity.

### Layers of Post-Transcriptional Control in the Circadian Timing System

Transcription is considered the prime mechanism driving rhythmic gene expression both in the core clockwork and in clock output. However, mRNA steady-state abundance is determined by the rates of both transcription and degradation and theoretical considerations have led to the conclusion that transcriptional rhythms are manifest in high amplitude mRNA cycling only when the mRNA has a sufficiently short half-life (Wuarin et al., 1992; Lück et al., 2014).

Enhancer trapping using a promoter-less luciferase reporter in *Arabidopsis* unveiled that one third of the genome is under transcriptional control by the clock (Michael and McClung, 2003). However, the *LHCB1^∗^3* (*LIGHT HARVESTING CHLOROPHYLL BINDING PROTEIN*) promoter is clock-regulated but transcript levels are constitutive, suggesting that changes in mRNA stability obscure rhythmic transcription (Millar and Kay, 1991). *CATALASE3* mRNA oscillations damp to a high level in constant darkness while *CATALASE3* promoter-driven luciferase activity still oscillates with an evening peak (Zhong et al., 1997; Michael and McClung, 2002). On the contrary, *NITRATE REDUCTASE* mRNA oscillates despite a time-of-day independent transcription rate (Pilgrim et al., 1993).

In mammals, about 10% of the transcripts in the liver undergo circadian oscillations (Akhtar et al., 2002). A comprehensive RNA-seq analysis uncovered that rhythms of only 22% of them are driven by *de novo* transcription (Koike et al., 2012). Furthermore, a recent transcriptome analysis with 2-h resolution around the clock found that 10% of the *N. crassa* transcriptome is reproducibly rhythmic at the mRNA level under normal growth conditions, and that the circadian clock may influence as much as 40% of the genome under other conditions (Hurley et al., 2014). A parallel high-throughput assay for timing of promoter activity using the luciferase reporter unveiled significant discordance between promoter activity and transcript oscillations. These discrepancies between clock-controlled transcription and oscillations in mRNA steady-state abundance pointed to additional levels of control impinging on clock-regulated transcripts and thus post-transcriptional regulation moved centre-stage in chronobiology.

Here, we discuss our current view on RNA-based regulation of gene expression in the *Arabidopsis* circadian timing system. Additionally, selected examples of RNA processing steps that have been shown to shape the daily pattern of transcripts in other model organisms including mammals, the fly *Drosophila melanogaster* and the bread mold *N. crassa* are presented for an integrated view. For a general survey of post-transcriptional regulation in these circadian clock systems readers are referred to comprehensive reviews (Crosthwaite, 2004; Harms et al., 2004; Keene, 2007; Kojima et al., 2011; Staiger and Green, 2011; Staiger and Köster, 2011; Zhang et al., 2011; Wang et al., 2013; Kojima and Green, 2014).

## Alternative Splicing in the Circadian System

In plants, our understanding of post-transcriptional regulation of circadian timekeeping is most advanced for alternative splicing. We begin by briefly describing key points of the mechanism, the players and the outcome of alternative splicing before turning to its relevance for circadian timekeeping in *Arabidopsis* and conclude by selected examples of alternative splicing in other model organisms of chronobiology.

### Regulation of Alternative Splicing

**FIGURE 3:**
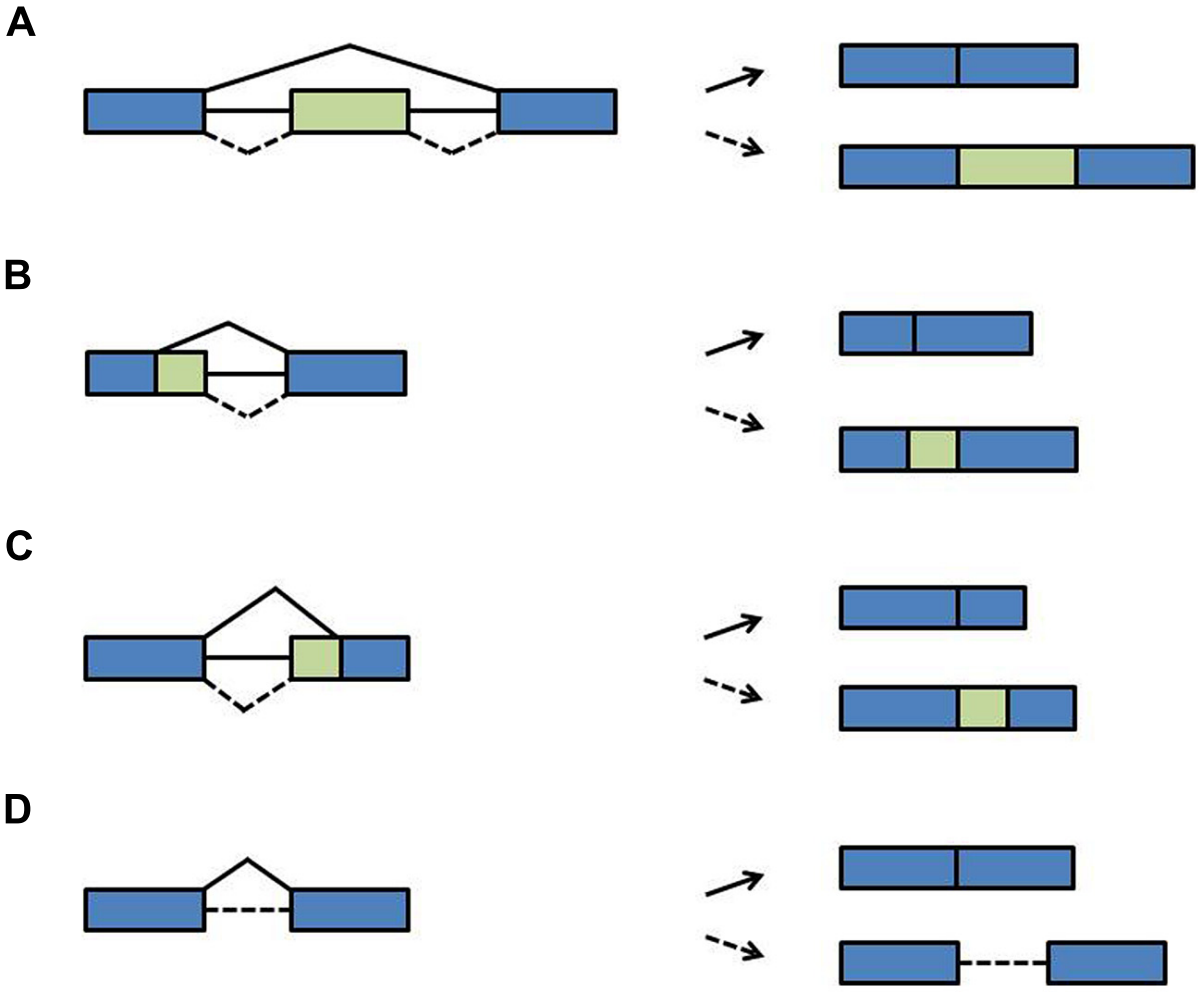
**Types of alternative splicing events. (A)** Exon skipping **(B)** alternative 5′ splice site **(C)** alternative 3′ splice site **(D)** intron retention. Blue boxes: exons; green boxes: alternative exons; lines: introns; solid diagonal lines: constitutive splicing events; broken diagonal lines: alternative splicing events.

During pre-mRNA splicing, introns are excised and the flanking exons are joined. However, not every splice site is used each time a pre-mRNA is processed. Rather, through the variable use of splice sites exonic sequences can be lost or intronic sequences can remain in the mRNA, designated as alternative splicing. During exon skipping, exons are removed together with their flanking introns (**Figure 3A**). The use of alternative 5′ splice sites or alternative 3′ splice sites causes variable portions of introns to be removed and variable portions of exons to remain in the mRNA (**Figures 3B,C**). During intron retention, introns can stay in the pre-mRNA (**Figure 3D**). Due to this variation in splicing patterns the corresponding proteins can be composed of distinct domains and thus have different functions (Nilsen and Graveley, 2010; Carvalho et al., 2012; Syed et al., 2012; Reddy et al., 2013). This tremendously increases the coding capacity of the genome. At the RNA level, alternative splice isoforms can have a different inventory of *cis*-regulatory sequence motifs and thus be differentially recognized by RNA-binding proteins or miRNAs. Alternative splice isoforms can also be identified as being “aberrant” and targeted for degradation. For example, if intronic sequences are retained, the open reading frame (ORF) may terminate at a premature termination codon (PTC). Such PTCs are recognized by the nonsense-mediated decay (NMD) pathway, a surveillance mechanism that eliminates aberrant transcripts (Arciga-Reyes et al., 2006; Isken and Maquat, 2008). Through linkage with NMD, alternative splicing can lead to quantitative changes in overall transcript levels (McGlincy and Smith, 2008; Nicholson and Mühlemann, 2010). Of note is that the NMD pathway has recently been shown to contribute to innate immunity in plant-pathogen-interaction and thus may have more widespread physiological roles (Gloggnitzer et al., 2014).

Pre-mRNA splicing is executed by a high molecular weight machine in the cell, the spliceosome. The main components of the spliceosome are five ribonucleoprotein (RNP) complexes with specific RNAs designated the U-rich small nuclear RNAs (U snRNAs). U1, U2, U4, and U5 assemble with the Sm proteins B/B’, D1, D2, D3, E, F, and G to form U snRNPs. The U6 snRNP contains the related LSM2 (Like-Sm2) to LSM8 proteins (Tharun, 2009; Golisz et al., 2013).

The decision whether a particular splice site is used is influenced by additional RNA-binding proteins that interact with distinct motifs in the pre-mRNAs to favor or inhibit the recruitment of the spliceosome to neighboring alternative splice sites (Reddy et al., 2012, 2013; Kornblihtt et al., 2013). These regulators are mainly serine/arginine-rich (SR) proteins or heterogeneous nuclear ribonucleoparticle proteins (hnRNPs). The SR proteins contain one or two RRMs as well as a domain with a high proportion of arginine/serine repeats (Reddy, 2004; Barta et al., 2010). The hnRNPs are a diverse class of RNA-binding proteins with one or multiple RRMs or RNA binding domains of the K homology (KH) motif type, originally found in human hnRNP K (Wachter et al., 2012). Numerous components involved in pre-mRNA splicing have been identified in *A. thaliana*, based on homology to yeast and mammalian sequences (Koncz et al., 2012; Reddy et al., 2013).

### Alternative Splicing in the Core Clockwork

**FIGURE 4:**
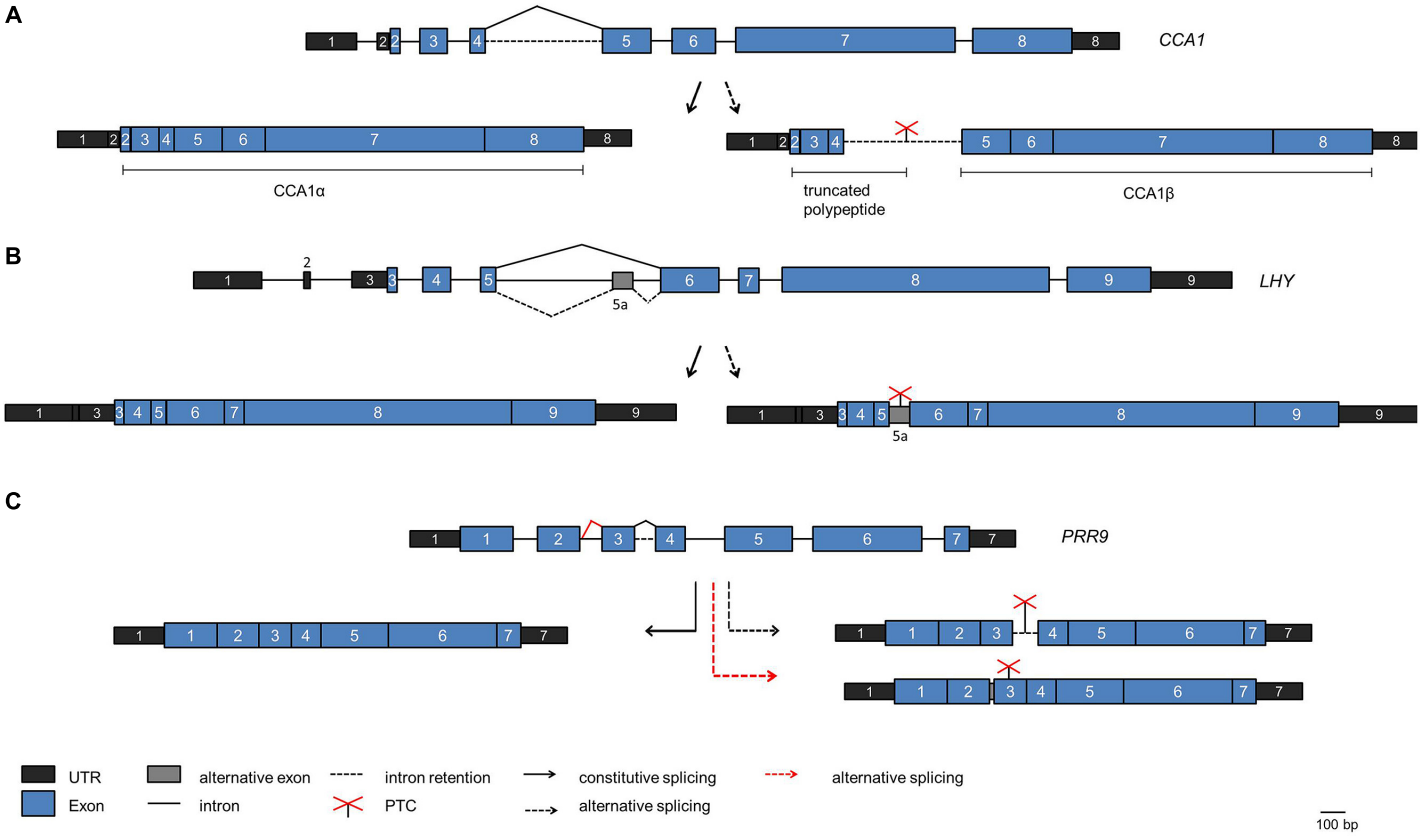
**Alternative splicing events in core clock genes**. The scheme of the genes are shown on the top of each panel, the fully spliced isoforms encoding the full length proteins are indicated on the left side and alternative splice isoforms are indicated on the right side. **(A)** *CCA1*. The fully spliced isoform encoding full length CCA1 [designated CCA1α by Seo et al. (2012)] is indicated on the left. The splice isoform with intron 4 retained is shown on the right. It is predicted to encode a polypeptide terminating at the PTC within intron 4, thus comprising only the DNA-binding Myb domain, or the CCA1β polypeptide comprising only the dimerization domain (Seo et al., 2012). **(B)** *LHY*. The splice isoform with alternative splicing at intron 5 leading to inclusion of exon 5a including a PTC is shown on the right. **(C)** *PRR9*. The splice isoform retaining intron 3 including a PTC is shown on the right (black broken arrow). The spliced isoform with additional eight nucleotides at exon 2 due to the use of an alternative 5′ splice site entailing a frame shift is shown on the right at the bottom (red broken line). This splice isoform is elevated in *prmt5*.

Whole transcriptome sequencing has uncovered a prominent role of alternative pre-mRNA splicing in the plant circadian system (Sanchez et al., 2011; Syed et al., 2012; Henriques and Mas, 2013; Staiger and Brown, 2013; Cui et al., 2014). For the core clock gene *CCA1* an alternative splice isoform was found to increase upon exposure of the plants to high light and decrease upon exposure to cold (Filichkin et al., 2010). This splice isoform retains intron 4, the long intron following the Myb-domain encoding exons, and thus can give rise to a truncated protein due to a PTC (**Figure 4A**). The splicing factor SR45 is able to bind to the intron *in vitro*, suggesting that it may function in alternative splicing of intron 4 (Filichkin et al., 2015).

A targeted search for alternative splice isoforms of the *Arabidopsis* clock genes using a high resolution Reverse transcription-PCR based alternative splicing panel uncovered that alternative splicing is widespread in the core clock (James et al., 2012). For *LHY*, an alternative splice isoform containing an alternative exon was found due to alternative splicing at the long intron 5 (that corresponds to intron 4 in the related *CCA1* transcript; **Figure 4B**). This splice isoform accumulates when plants are shifted to low temperature. Because it contains a PTC and is a substrate for the NMD pathway the production of full length LHY protein is precluded and thus LHY protein levels decline at low temperature (James et al., 2012). In contrast, *CCA1* intron 4 retention decreases at low temperature, and the *CCA1* transcript transiently shows a higher and broader peak upon shift to low temperature. These data suggest that alternative splicing may adjust the core oscillator to low temperatures, and the differential behavior of CCA1 and LHY in this response may contribute to the overlapping but not redundant action of these two proteins in the clock mechanism.

Subsequently, autoregulation of CCA1 has been proposed to underlie this low temperature response. The *CCA1* alternative splice isoform retaining intron 4 including a PTC accumulates at low temperature, as described above, and can produce a polypeptide comprising only the N-terminal MYB domain (**Figure 4A**). This transcript has been predicted to produce a protein that consists of the C-terminal dimerization domain without the DNA-binding MYB domain, designated CCA1β (**Figure 4A**; Seo et al., 2012). Upon targeted over-expression in transgenic plants CCA1β interferes with complex formation of the full length CCA1α protein both with itself and with LHY and thus with their function as transcriptional repressors in the core clock. Indeed, over-expression of CCA1β from a constitutive promoter leads to a short period phenotype, as observed in *cca1 lhy* mutants, consistent with CCA1β acting as a dominant negative inhibitor. It remains to be demonstrated whether such a reinitiation of translation downstream of an ORF occurs *in planta* to produce the CCA1β protein.

Similar to CCA1 and LHY, PRR7, and PRR9 are thought to act partially redundantly in the clock. For *PRR7* non-functional alternative splice isoforms transiently accumulate to substantial levels upon exposure to low temperatures and thus would lead to reduced PRR7 levels. In contrast, *PRR9* transiently increases at low temperatures, but the *PRR9* alternative splicing events that lead to non-functional transcripts due to intron 3 retention or inclusion of eight nucleotides at the end of exon 2 are not influenced by temperature (**Figure 4C**; James et al., 2012). This points to differences in the regulation of this pair of clock proteins. *TOC1*/*PRR1* and *PRR5* are also alternatively spliced to PTC-containing isoforms at low temperatures.

Alternative splicing of clock genes has also been observed upon exposure to high temperatures, e.g., for the *LUX*, *LOV KELCH PROTEIN 2* and *TIME FOR COFFEE* (*TIC*) transcripts but the physiological consequences for clock function have not been addressed (Filichkin and Mockler, 2012).

For *PRR7* and *LHY* rapid changes in alternative splicing patterns in response to red light activation of the phytochrome photoreceptor have been uncovered (Shikata et al., 2014). Given the role of phytochrome in mediating light input (Fankhauser and Staiger, 2002; Millar, 2004), it is conceivable that such changes in alternative splicing may impact light entrainment of the clock.

### Alternative Splicing in Clock Output

Currently it is estimated that alternative splicing affects more than 60% of all intron-containing genes in *Arabidopsis* (Marquez et al., 2012). The functional relevance of most of the alternative splice isoforms remains to be demonstrated (Carvalho et al., 2012). A differential function was found for two RIBULOSE-1,5-BISPHOSPHATE CARBOXYLASE ACTIVASE (RCA) protein variants encoded by splice isoforms. The *RCA* transcript undergoes circadian oscillations in steady-state abundance as well as circadianly regulated alternative splicing (Sanchez et al., 2010). A short alternative splice isoform encodes a protein that acts independent of light, and a long alternative splice isoform encodes a protein isoform regulated by light (Zhang et al., 2002). Alternative splicing of the mRNA isoform that encodes the light-regulated protein increases during the day (Sanchez et al., 2010).

The use of whole-genome tiling arrays for transcript profiling around the clock unveiled circadian rhythms in the steady-state level of numerous introns (Hazen et al., 2009). In cases where these retained introns are embedded in rhythmically expressed genes and oscillate in phase with their surrounding exons, the retained introns should lead to truncated protein variants.

### A Circadian Feedback Loop based on Alternative Splicing and NMD

**FIGURE 5:**
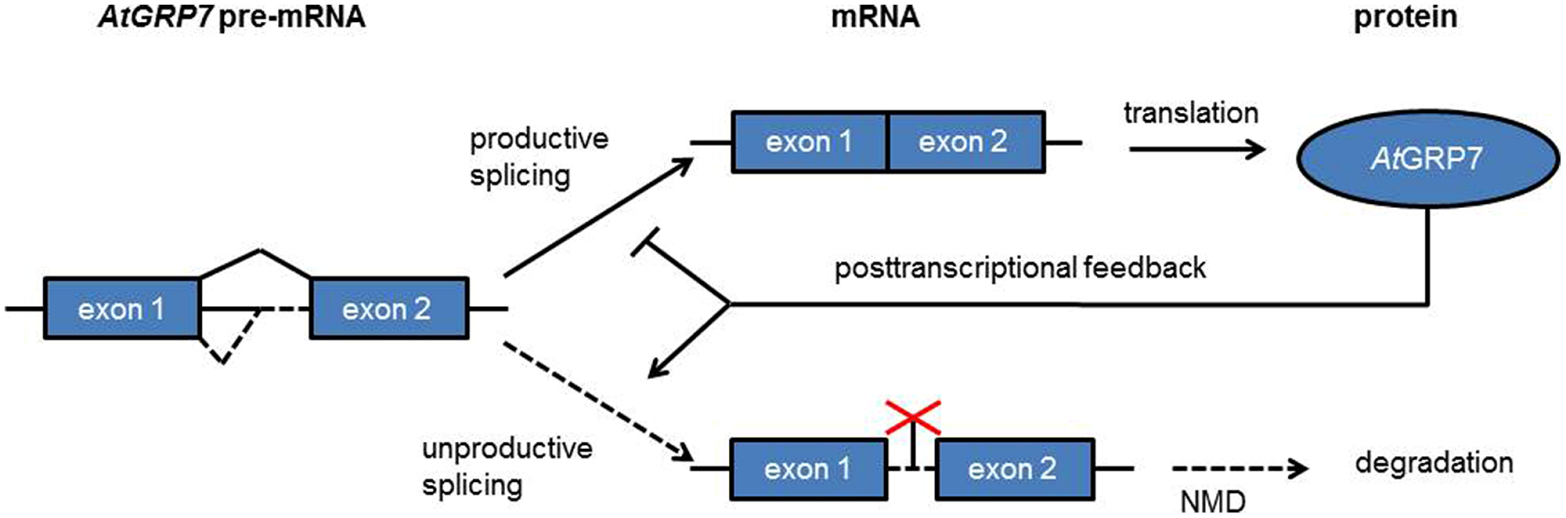
**A clock-regulated negative autoregulatory circuit based on alternative splicing and NMD**. In response to increasing *At*GRP7 levels the use of the cryptic intronic 5′ splice site is favored, leading to unproductive splicing with the splice isoform being degraded via NMD.

*At*GRP7 (*A. thaliana* glycine-rich RNA binding protein 7) and *At*GRP8 are clock-regulated RNA binding proteins which peak at the end of the day. Both *At*GRP7 and *At*GRP8 negatively autoregulate through alternative splicing (**Figure 5**). A transcript isoform retaining part of the intron including a PTC is generated that rapidly decays via NMD (Staiger and Heintzen, 1999; Schöning et al., 2008). Upon mutation of a conserved arginine residue in the RRM the *in vitro* and *in vivo* RNA binding activities as well as the negative autoregulation of *At*GRP7 are lost (Schöning et al., 2007; Köster et al., 2014a; Leder et al., 2014). *At*GRP7 and *At*GRP8 thus represent two clock-regulated feedback circuits that additionally cross-regulate via alternative splicing and NMD. These were the first examples of feedback loops based on post-transcriptional regulation in the circadian system. *At*GRP7 and *At*GRP8 in turn regulate steady-state abundance or alternative splicing of several transcripts which undergo circadian oscillations themselves, suggesting that the *At*GRP7/*At*GRP8 feedback loops pass timing information from the core oscillator to clock output (Rudolf et al., 2004; Streitner et al., 2010, 2012; Schmal et al., 2013).

Apart from circadian rhythms, *At*GRP7 has been shown to be involved in the low temperature response, response to oxidative stress, flowering time control and pathogen defense (Kim et al., 2007b, 2008; Schmidt et al., 2010; Nicaise et al., 2013; Hackmann et al., 2014; Löhr et al., 2014). This suggests that it may exert widespread post-transcriptional control in the cell and integrate responses to external stimuli with endogenous timing cues.

### A Defective Clock in Mutants of Candidate Splicing Components

A long period circadian phenotype has been observed in mutants deficient in PROTEIN METHYLTRANSFERASE 5 (PRMT5; Sanchez et al., 2010, 2011). PRMT5 is a type II protein arginine methyltransferase that symmetrically dimethylates arginine side chains, i.e., deposits a methyl group on either one of the two terminal guanidino nitrogens. PRMT5 acts upon a broad spectrum of substrates that can be detected by antibodies against symmetrically dimethylated arginine in wild type plants, but not in the *prmt5* mutant. Among those are numerous proteins involved in RNA processing including AtSmD1, AtSmD3, and LSM4 (Deng et al., 2010). In *Arabidopsis*, a complex of LSM2 to LSM8 complex is part of U snRNPs and thus functions in pre-mRNA splicing (Perea-Resa et al., 2012). In *prmt5*, *PRR9* levels are greatly elevated compared to wild type plants. As *PRR9* over-expression leads to a short period of the clock, altered PRR9 steady-state abundance seemed unlikely to cause the *prmt5* long period phenotype (Matsushika et al., 2002). However, the *PRR9* transcript was aberrantly spliced in *prmt5*. An alternative splice isoform with a PTC due to the use of an alternative 5′ splice site at intron 2 accumulate at the expense of the functional isoform (**Figure 4B**). This suggests that the long period in *prmt5* can be partly attributed to aberrant *PRR9* splicing. When the *prmt5* mutant was analyzed on tiling arrays, a global impact of PRMT5 on alternative splicing was found. The use of the high resolution alternative splicing panel then unveiled that PRMT5 function is required in particular to activate weak 5′ splice sites (Sanchez et al., 2010).

Subsequently, mutants defective in the PRMT5 substrate LSM4 were also linked to circadian regulation. The *lsm4* mutant shows long period oscillations of gene expression as well as aberrant splicing of several clock genes (Perez-Santángelo et al., 2014). The *lsm5* mutant, previously identified because of its hypersensitivity to abscisic acid and thus named *sad1* (*supersensitive to abscisic acid 1*) also led to long period leaf movement rhythms (Perez-Santángelo et al., 2014). The expression of several clock genes is altered in *lsm5*. Furthermore, there is an increase in *TOC1* intron 4 retention, similar to wild-type plants exposed to low temperature conditions, and intron 2 is retained in *CCA1*. A genome-wide analysis of both mutants uncovered a more widespread role of LSM4 in the control of alternative splicing. Notably, the *LSM5* transcript undergoes circadian oscillations itself. Such a clock regulation of splicing regulators like LSM5 or *At*GRP7 may serve as a means to coordinate time-of-day dependent changes in splicing of a cohort of target genes (Staiger, 2001; Perez-Santángelo et al., 2013).

Mutations in two other splicing factors, Ski-interacting protein (SKIP) and SPLICEOSOMAL TIMEKEEPER LOCUS 1 (STIPL1), led also to a long period of the clock (Jones et al., 2012; Wang et al., 2012). The yeast and human SKIP counterparts have a demonstrated role as splicing factors (Gahura et al., 2009). AtSKIP associates with the splicing factor SR45 and in the *skip-1* mutant, alternative splicing of *PRR7* and *PRR9* is defective, contributing to the long period phenotype (Wang et al., 2012). STIPL1 encodes a homolog of TUFTELIN-INTERACTING PROTEIN 11 (TFIP11) in humans and Ntr1p in yeast involved in spliceosome disassembly (Tannukit et al., 2009; Jones et al., 2012). The altered expression of *CCA1*, *LHY*, *PRR9*, *GI*, and *TOC1* caused by the aberrant splicing is likely to contribute to the circadian defects in the *stipl1* mutant. The mechanisms of how the *Arabidopsis* proteins impact splicing of their targets remain to be described.

In mice, a very interesting rhythmic and light-induced alternative splicing event was described for the splicing factor U2AF26, a homolog of the small U2 auxiliary factor (U2AF) subunit U2AF35 involved in recognition of the 3′ splice site (Preußner et al., 2014). Through this alternative splicing events, translation of U2AF26 extends into the 3′ untranslated region (3′ UTR), generating a C-terminal extension of the ORF. This additional domain shows homology to the *Drosophila* clock protein Timeless, an interaction partner of Period. U2AF26-deficient mice show nearly arrhythmic Period1 protein levels and aberrant mRNA cycling in peripheral clocks. Moreover, lack in U2AF26 leads to increased phase advance in response to alterations in the environmental light–dark cycles. These data suggest that light induced U2AF26 alternative splicing serves to limit *Period1* induction in response to changes in ambient light and thus is involved in entrainment (Preußner et al., 2014).

## RNA Stability

Eukaryotic mRNAs vary widely in their stabilities and mRNA turnover is exquisitely regulated (Houseley and Tollervey, 2009). The first step in mRNA degradation is the removal of the poly(A) tail, followed by exoribonuclease digestion from the 5′ end after removal of the cap structure or digestion from the 3′ end by the exosome, a multi-subunit machinery for RNA degradation in eukaryotes.

It has long been predicted that changes in mRNA half-life across the circadian cycle contribute to circadian transcript oscillations (So and Rosbash, 1997). In *Arabidopsis*, a suite of clock-controlled transcripts were identified in an approach to globally identify short-lived transcripts using DNA microarrays (Gutierrez et al., 2002). For two of them, *CCR-LIKE* (*CCL*) and *SENESCENCE ASSOCIATED GENE 1*, mRNA stability changes across the day. The changes in *CCL* mRNA stability continue under free-running conditions, indicating that they are controlled by the circadian clock (Lidder et al., 2005). Furthermore, degradation of these mRNAs is mediated by the downstream (DST) element first identified in the 3′ UTR of the auxin inducible SMALL AUXIN-UP RNAs and shown to destabilize mRNA (Newman et al., 1993). Of note, disruption of the DST-mediated RNA decay pathway leads to circadian defects (Lidder et al., 2005). Components of the DST pathway have not yet been reported.

For the core clock gene *CCA1*, a dependence of transcript stability on light quality has been found (Yakir et al., 2007). The *CCA1* mRNA is relatively stable in the dark but has a short half-life in the light. The light-dependent *CCA1* mRNA degradation in combination with light-regulated *CCA1* transcription has been implicated in entrainment of the clock. Again, little is known about mechanisms such as, for example, RNA-binding proteins that regulate the access of nucleases, e.g., through steric hindrance or conformational changes in the mRNA depending on the light quality.

In mammals, changes in RNA stability contribute to oscillations of the core clock genes *Cry1*, *Per1*, *Per2,* and *Per3*, with a higher stability during the upswing and a lower stability during the downswing. Several cellular RNA-binding proteins including hnRNP D and hnRNP I, also known as the polypyrimidine tract binding protein PTB, have been shown to bind to the 3′ UTRs of clock transcripts in a circadian phase dependent manner, entailing their degradation (Kwak et al., 2006; Woo et al., 2009, 2010; Lee et al., 2014; Kim et al., 2015).

In *N. crassa*, the exosome is involved in regulation of several rhythmic transcripts (Guo et al., 2009). For example, downregulation of the *Neurospora* ortholog of RRP44, the 3′→5′ exonuclease subunit of the exosome, leads to enhanced *frq* mRNA stability, higher *frq* levels and a longer period of *frq* mRNA oscillations. Because FRH interacts with the RRP44 ortholog, FRQ, FRH and the exosome are part of a post-transcriptional negative feedback loop interlocked with the clock transcriptional feedback loop that regulates WCC activity. The *rrp44* transcript itself is clock-controlled suggesting that time-of-day-dependent exosome activity may play a wider role in circadian regulation.

In the green algae *Chlamydomonas reinhardtii* knockdown of XRN1, a 5′→3′ exoribonuclease leads to low amplitude and rapid dampening of the bioluminescence rhythm (Matsuo et al., 2008). XRN1 has been shown to interact with the C3 subunit of the RNA-binding protein CHLAMY1 that is required for correct period and phase of circadian rhythms (Dathe et al., 2012).

## Alternative Polyadenylation and Poly(A) Tail Length

Processing at the mRNA 3′ end comprises pre-mRNA cleavage at the poly(A) site followed by the addition of tracts of adenosines (Xing and Li, 2011). This poly(A) tail influences both mRNA stability and translation. A dedicated group of RNA-binding proteins, the poly(A) binding proteins, bind to the poly(A) tail and additionally interact with the translation initiation factor eIF4G that in turn interacts with the Cap binding protein eIF4E, thus bending the mRNA into a circle. This enables translational control by the poly(A) tail.

New insights into polyadenylation in *Arabidopsis* came from implementation of direct RNA sequencing, a single molecule technique where native mRNA is used as the template (Sherstnev et al., 2012). This allowed the determination of the site of RNA cleavage and polyadenylation without errors cause by aberrant reverse transcription or PCR amplification during library generation and unveiled a widespread heterogeneity in 3′ ends through alternative polyadenylation (Sherstnev et al., 2012). Such variation in the length of the 3′ UTRs can have functional significance for the resulting transcript isoforms, e.g., due to the presence of different regulatory motifs or miRNA binding sites in 3′ UTRs of different length. The functional consequences of the usage of the alternative polyadenylation sites for core clock genes and genes of clock output remain to be resolved.

In the cytoplasm, poly(A) tail shortening from the 3′ end is catalyzed by deadenylases. This variation in poly(A) tail length can affect mRNA stability. A connection of variation in poly(A) tail length with circadian rhythms has not been addressed in plants.

In mammals, daily fluctuations in poly(A) tail size have initially been observed for the mRNA encoding the neuroactive peptide vasopressin that is synthesized within the suprachiasmatic nuclei and displays rhythmic concentration changes in the cerebrospinal fluid (Robinson et al., 1988). More recently, 2.5% of transcripts in mouse liver turned out to undergo circadian oscillations in poly(A) tail length in a genome-wide poly(A)denylome analysis using microarrays (Kojima et al., 2012). Interestingly, this variation in poly(A) tail length rather than variation in mRNA steady-state abundance correlated with rhythms in protein abundance, suggesting that the rhythmic variation in the length of the poly(A) tail leads to rhythmic translation.

One of the deadenylases in mammals, Nocturnin, is rhythmically expressed (Wang et al., 2001). Mice deficient for Nocturnin have defects in lipid homeostasis and response to glucose, suggesting that Nocturnin mediates post-transcriptional regulation of metabolic events by the circadian clock.

## Translational Control

Changes in translation efficiency allow a rapid adjustment in the proteome without prior regulation of transcription or RNA processing. Regulation at the level of translation initiation is a topic intensely studied in plants (Roy and von Arnim, 2013). In particular, upstream ORF (uORFs) located 5′ to a reading frame can alter the usage of its start codons. When such uORFs are longer than 25 codons they inhibit translation of the downstream ORF (Nyiko et al., 2009; Roy et al., 2010). About a third of all *Arabidopsis* genes harbor uORFs but few of them have been functionally studied (Kim et al., 2007a). Alternative splicing of introns in the 5′ UTR can affect the inventory of uORFs. Moreover, miRNAs can affect translation of their target mRNAs (Brodersen et al., 2008; Li et al., 2013).

Several studies in *Arabidopsis* indicate that protein levels often do not follow rhythmic mRNA levels (Piques et al., 2009; Baerenfaller et al., 2012). In rice seedlings, a number of proteins display oscillations at the protein level that do not correlate with mRNA rhythms (Hwang et al., 2011). On the one hand, such observations point to rhythms in translation. On the other hand, cycling of proteins made from rhythmic mRNAs can also be blunted through long half-lives. In tomato, *in vivo* labeling with 35S methionine uncovered circadian oscillations of the translation rate of the photosystem II light harvesting complex polypeptide that closely correlate with the transcript oscillations (Riesselmann and Piechulla, 1992). In contrast, no oscillation was detected at the protein level, perhaps due to the low turnover of this membrane protein.

In *Arabidopsis*, translation of the core clock gene *LHY* has been shown to be influenced by light (Kim et al., 2003). Thus, when LHY transcript levels fall after the dawn peak, light promotes *LHY* translation. This simultaneous translational induction and transcriptional repression has been suggested to sharpen the LHY protein peak.

Clearly, a more widespread inventory of changes in translation would provide insights into clock-control over translation. Comparing the pool of mRNAs associated with polysomes to the mRNAs not associated with polysomes serves as an indication for active translation. Such a global translatome profiling across the circadian cycle would allow conclusions about changes in the translation status of each transcript in the course of the day (Missra and von Arnim, 2014).

In mammals, dedicated RNA-binding proteins have been identified that control translation of clock genes. The mLARK protein binds to the 3′ UTR of *Per1* and boosts PER1 protein levels, most likely through stimulation of translation (Kojima et al., 2007). Additionally, hnRNPQ rhythmically binds to the *Per1* 5′ UTR to stimulate its translation in a time-of-day dependent manner (Lee et al., 2012).

Notably, as many as 50% of the proteins that cycle in liver are translated from constitutively expressed mRNAs (Reddy et al., 2006). More recently, it has been observed that the clock exerts also a widespread control of the translation apparatus through coordinated transcription of translation initiation factors, ribosomal proteins, and rRNAs (Jouffe et al., 2013). Additionally, distinct signaling pathways impinging on translation initiation factors are rhythmically activated.

In *C. reinhardtii*, the RNA-binding protein CHLAMY1 regulates translation of output genes. CHLAMY1 binds, in a circadian manner, to transcripts with UG repeats in their 3′ UTR (Zhao et al., 2004). Among these transcripts is NITRITE REDUCTASE (Waltenberger et al., 2001). Consistent with a role for CHLAMY1 as a translational repressor, the activity of NITRITE REDUCTASE in reciprocal to the levels of CHLAMY1 C1 and 3 subunits.

## Non-Coding RNAs

### microRNAs

Plant miRNAs regulate a wide range of mRNAs predominantly by mRNA cleavage and subsequent degradation but also via inhibition of translation (**Figure 1**; Rogers and Chen, 2013). In *Arabidopsis*, a suite of miRNAs were interrogated for rhythmic expression. miR171, miR398, miR168, and miR167 oscillate diurnally but are not under clock-control (Sire et al., 2009). In contrast, the precursors of miR157A, miR158A, miR160B, and miR167D are clock-controlled (Hazen et al., 2009). Whether these daily fluctuations in expression have functional consequences for their targets has not been addressed. Notably, clock-regulated RNA-binding protein *At*GRP7 impacts processing of several miRNA precursors (Köster et al., 2014b).

In mammals, rhythmic miRNA expression has been widely observed. Several clock genes are targets of miRNAs, and miRNAs have been implicated in the regulation of period length and light resetting of the clock (Nagel et al., 2009). Recently, miR122 was shown to control the expression of the deadenylase nocturnin, another post-transcriptional regulator in the circadian system (Kojima et al., 2010).

### Natural Antisense Transcripts

*Arabidopsis* contains a large number of convergently overlapping gene pairs that can give rise to natural antisense transcripts (NATs) which might act as regulators of the sense gene (Zubko et al., 2011). Using tiling arrays, rhythmic NATs have been detected for 7% of the protein coding genes (Hazen et al., 2009). Among these are the oscillator genes *LHY*, *CCA1*, *TOC1*, *PRR3*, *PRR5*, *PRR7,* and *PRR9*. The functional significance for the oscillator mechanism has not been addressed.

A prime example of antisense RNA regulation in circadian timekeeping is the *N. crassa frq* locus that gives rise to a long non-coding antisense RNA *qrf* oscillating in antiphase to *frq* (Kramer et al., 2003). Light-dependent *qrf* expression is involved in resetting of the clock (Xue et al., 2014). Moreover, *frq* transcription and *qrf* transcription are mutually inhibitory, resulting in the antiphasic *frq* and *qrf* oscillations. Antisense *Per1* transcripts that cycle in antiphase to *Per1* have been detected in mouse liver and in silkmoth, suggesting that such pairs of rhythmic antisense RNAs may also play a role in circadian clocks (Sauman and Reppert, 1996; Koike et al., 2012; Menet et al., 2012).

As the number of identified ncRNAs including ncRNAs and siRNAs is increasing, it is conceivable that more ncRNAs will be found to fulfill a role in regulating circadian gene expression.

### The Epitranscriptome

Modification of cellular components by methylation is mostly known for DNA and proteins. Methylation generally depends on the availability of *S*-adenosylmethionine (SAM) that donates the methyl group and the concentration of *S*-adenosylhomocysteine (SAH), a by-product that is a competitive inhibitor of the methylation. The drug 3-deazaadenosine inhibits SAH hydrolysis and thus indirectly inhibits methylation though accumulation of the competitive inhibitor (Chiang, 1998).

The role of histone methylation in clock gene transcription has been established in *Arabidopsis* (Malapeira et al., 2012; Song and Noh, 2012). The role of non-histone protein methylation in the clock is obvious from the pervasive effect of the *prmt5* mutant on the pace of *Arabidopsis* clock (Hong et al., 2010; Sanchez et al., 2010).

In contrast to DNA and protein methylation, the physiological role of mRNA modification by methylation of nucleobases is less well understood. Nevertheless, the importance of RNA methylation including methylation of adenine (m^6^A) has recently been recognized in plants (Bodi et al., 2012). Impaired m^6^A methylation affects embryonic development and leads to aberrant growth phenotypes in adult plants. Within transcripts, m^6^A was found predominantly about 150 nucleotides upstream of the polyadenylation site.

RNA methylation has recently been shown to affect the mammalian clock (Fustin et al., 2013). Treatment of human cells with 3-deazaadenosin led to global changes in gene expression. The gene ontology category “rhythmic processes” ranked fourth among significantly affected processes, suggesting an exquisite sensitivity of the circadian clock to imbalanced methylation. Among the upregulated genes were RNA processing factors including RNA m^7^G cap methylases, m6A demethylases, RNA methylases, and splicing factors. RNA immunoprecipitation using an antibody against m^6^A then unveiled the presence of m6A in many clock gene transcripts. Inhibition of m6A RNA methylation by knockdown of the methyltransferase Mettl3 led to a long period of *Per2* driven luciferase activity and locomoter activity. The processing of clock genes including *Per2* was delayed which may slow down the speed of the clock.

## Conclusion

Ample evidence has accumulated for discordances between rhythms in promoter activity and rhythms in mRNA levels on the one hand and mRNA and protein rhythms on the other hand, respectively. In particular, the importance of correct alternative pre-mRNA splicing in the *Arabidopsis* clock has been recognized either through aberrant clock function in splicing factor mutants or the appearance of specific alternative splice isoforms of clock genes. In the future, the recent establishment of STABLE ISOTOPE LABELING by amino acids in cell culture (SILAC) for *Arabidopsis* seedlings will allow quantitative proteomics and identification of predicted polypeptides corresponding to alternative splice isoforms (Lewandowska et al., 2013). A next logical step is to prove the relevance of alternative splice isoforms by testing their association with polysomes and through complementation of mutants with forced isoform expression. Furthermore, the development of robust RNA immunoprecipitation protocols provides an entré to identify direct targets of candidate splicing factors (Terzi and Simpson, 2009; Köster et al., 2014a).

**FIGURE 6:**
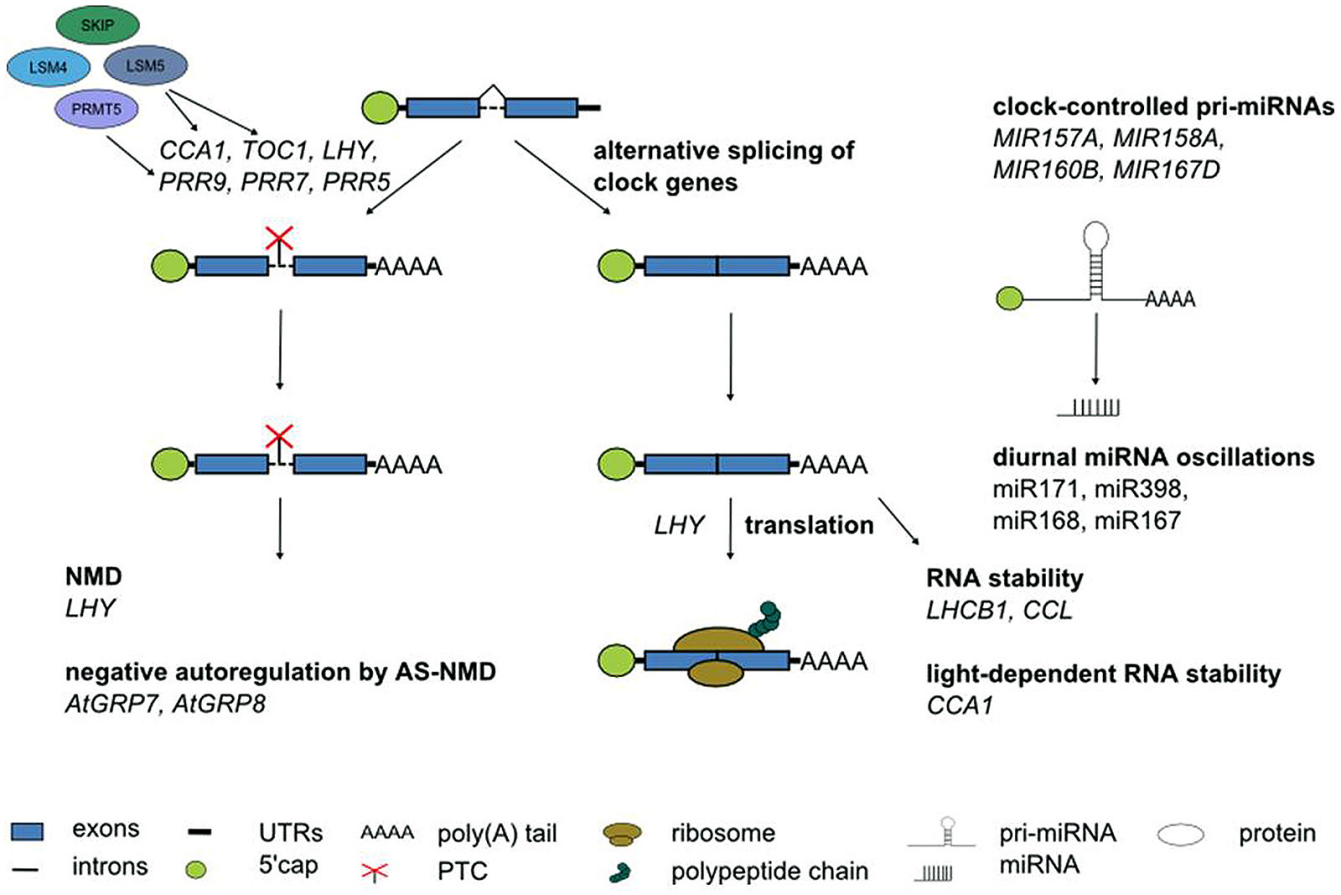
**Post-transcriptional processes in the *Arabidopsis* circadian system**. Numerous core clock genes undergo alternative splicing, in particular in response to temperature changes. The spliceosomal Lsm4 and Lsm5 proteins control alternatives splicing. PRMT5 affects alternative splicing of core clock genes likely through modification of snRNP proteins and splicing factors. Alternative splice isoforms of clock genes containing a PTC can undergo NMD. The RNA-binding proteins *At*GRP7 and *At*GRP8 are part of a clock-controlled post-transcriptional feedback loop based on alternative splicing and NMD. Translation of the core clock component *LHY* is regulated. The stability of *LHCB1* and *CCL* is time-of-day dependent. *CCA1* stability depends on the light quality. Some miRNA show diurnal oscillations. Several pri-miRNAs undergo clock-controlled oscillations. See text for further details.

Although alternative splicing undoubtedly is the layer of post-transcriptional regulation currently understood in most detail in *Arabidopsis*, it would be premature to assume that it is the most important one. Other RNA processing steps clearly contribute to shaping the circadian transcriptome (**Figure 6**). This is evident from the anecdotal reports on regulated stability of a few circadian genes, oscillations of a handful of miRNAs or translational regulation of clock genes summarized here. In particular, in the light of fundamental differences in the mechanism of pri-miRNA processing and in target mRNA regulation by miRNAs between animals and plants it will be interesting to see the impact of miRNAs on rhythmic gene expression programs. Novel developments in high throughput techniques combined with more powerful bioinformatics pipelines will help to further shift the focus from individual gene expression patterns to genome-wide impact of these regulatory events. This will advance our knowledge on the importance of other RNA processing steps for the circadian system also in plants, as it has been the case in animals.

## Conflict of Interest Statement

The authors declare that the research was conducted in the absence of any commercial or financial relationships that could be construed as a potential conflict of interest.
